# Relation of Automated Body Condition Scoring System and Inline Biomarkers (Milk Yield, β-Hydroxybutyrate, Lactate Dehydrogenase and Progesterone in Milk) with Cow’s Pregnancy Success

**DOI:** 10.3390/s21041414

**Published:** 2021-02-18

**Authors:** Ramūnas Antanaitis, Vida Juozaitienė, Dovilė Malašauskienė, Mindaugas Televičius, Mingaudas Urbutis, Walter Baumgartner

**Affiliations:** 1 Large Animal Clinic, Veterinary Academy, Lithuanian University of Health Sciences, Tilžės str 18, LT-47181 Kaunas, Lithuania; dovile.malasauskiene@lsmuni.lt (D.M.); mindaugas.televicius@lsmuni.lt (M.T.); mingaudas.urbutis@lsmuni.lt (M.U.); 2 Department of Animal Breeding, Veterinary Academy, Lithuanian University of Health Sciences, Tilžės Str 18, LT-47181 Kaunas, Lithuania; vida.juozaitiene@lsmuni.lt; 3 University Clinic for Ruminants, University of Veterinary Medicine, Veterinaerplatz 1, A-1210 Vienna, Austria; walter.baumgartner@vetmeduni.ac.at

**Keywords:** In-line, automated body condition scoring system, biomarkers, biosensors

## Abstract

The aim of the current study was to evaluate the relation of automatically determined body condition score (BCS) and inline biomarkers such as β-hydroxybutyrate (BHB), milk yield (MY), lactate dehydrogenase (LDH), and progesterone (mP4) with the pregnancy success of cows. The cows (*n* = 281) had 2.1 ± 0.1. lactations on average, were 151.6 ± 0.06 days postpartum, and were once tested with “Easy scan” ultrasound (IMV imaging, Scotland) at 30–35 d post-insemination. According to their reproductive status, cows were grouped into two groups: non-pregnant (*n* = 194 or 69.0% of cows) and pregnant (*n* = 87 or 31.0% of cows). Data concerning their BCS, mP4, MY, BHB, and LDH were collected each day from the day of insemination for 7 days. The BCS was collected with body condition score camera (DeLaval Inc., Tumba, Sweden); mP4, MY, BHB, and LDH were collected with the fully automated real-time analyzer Herd Navigator™ (Lattec I/S, Hillerød, Denmark) in combination with a DeLaval milking robot (DeLaval Inc., Tumba, Sweden). Of all the biomarkers, three differences between groups were significant. The body condition score (BCS) of the pregnant cows was higher (+0.49 score), the milk yield (MY) was lower (−4.36 kg), and milk progesterone in pregnant cows was (+6.11 ng/mL) higher compared to the group of non-pregnant cows (*p* < 0.001). The pregnancy status of the cows was associated with their BCS assessment (*p* < 0.001). We estimated that cows with BCS > 3.2 were 22 times more likely to have reproductive success than cows with BCS ≤ 3.2.

## 1. Introduction

The novel digital equipment, such as multiple sensors, a data infrastructure, and data analytics, can be used to monitor animals or their environment [1]. Blood biomarkers are valuable indicators of animal health but not generally applicable commercially. They could provide a lot of information, especially because subclinical stages of diseases can be detected by biomarkers while the cow may appear completely healthy, showing no external signs of sickness. One alternative for blood biomarkers would be biomarkers in milk, which are easily acquired but are yet to be well characterized [2]. Biomarkers and parameters from herd management programs, such as plasma β-hydroxybutyrate (BHB) and body condition score (BCS) are used to diagnose subclinical ketosis, nonetheless BHB has been shown as a valuable indicator of displaced abomasum in dairy cows [3]. A strong positive statistically significant relationship has been found between BHB and the average milk yield within all groups of primiparous cows, although we found a statistically unreliable correlation between highest milk yield and BHB in multiparous and primiparous cows [4]. Elevated levels of BHB in blood have been associated with reduction in milk yield, impaired reproductive performance, and increased occurrence of metabolic disorders; hence, early detection of higher BHB levels is crucial [5]. For the detection of subclinical mastitis, a good indicator is measurement of inline lactate dehydrogenase (LDH) activity in milk, for it is both simple and cost effective with high sensitivity and specificity [6]. Larsen et al. [7] reported that the milk enzyme LDH performed equally with acute phase proteins and somatic cell count as inflammatory indicators of mastitis. 

Body condition score (BCS) is a method to estimate body fat reserves in cows [8]. Not only that, BCS is also important in farm management [9]. It was found that BCS at calving and its changes throughout lactation affect the health and fertility of high producing dairy cows [10]. Cow BCS monitoring is important because it is a measure for the fat reserves of a cow, also suitable in the assessment of feeding management. Despite its importance, BCS is currently a time-consuming manual task usually performed only by experts [8]. Therefore, technological advances have been made that offer aid in BCS estimation [5]. BCS is a useful method of monitoring relations among nutritional management, reproduction, and ketosis and aids in farm management decisions [11]. For an even better herd assessment, a fully automated inline LDH progesterone (mP4), and BHB analyzer, which can be combined with a milking robot, is available for purchase in the commercial market. Using its precise diagnostic technologies can improve our understanding of the current factors affecting the reproductive physiology of dairy cows. Not only does the system assist with reproductive management, but with the assessment of frequent mP4 data, it allows for the evaluation of luteal activity and its association with fertility [12]. DeLaval Corporate designed the first commercially available 3D BCS system based on image processing technologies [13].

We hypothesized that biomarkers, such as body condition score, β-hydroxybutyrate, milk yield, lactate dehydrogenase, and progesterone have a relationship with the reproductive success of cows. To test our hypothesis, the aim of the current study was to determine the relationship of these biomarkers with successful pregnancy in cows and to check if the biomarkers differed between pregnant and non-pregnant cows.

## 2. Materials and Methods

### 2.1. Location, Animals and Experimental Design

The research was carried out in accordance with the standards set by Animal Welfare and Protection of the Republic of Lithuania (No. 108-2728; 2012, No. 122-6126). The study approval number is PK016965. The study was performed on one dairy farm located at 55.10571, 24.24399. For this current study, 281 Lithuanian Black and White dairy cows were selected, which were being kept in a loose housing system with free-stalls. All estrus cycles of the cows were synchronized according to the OvSynch protocol. The animals were considered to be in estrus and ready for insemination when they exhibited: a progesterone alarm (registered by Herd navigator system), an increase in cows walking activity (registered by AMS), and one or more of the following signs of estrus described by Van Eerdenburg et al. [14]: standing to be mounted, mucous vaginal discharge, cajoling, restlessness being mounted but not standing, sniffing vagina of other cows, resting chin on other cows, mounting (or attempted mounting) of other cows, or mounting the head part of other cows. Their uterine tone was examined by rectal palpation. After 12 h from the beginning of estrus (which was determined by mP4 concentration from AMS), the cows were artificially inseminated. The pregnancies were tested once with ‘Easy scan’ ultrasound (IMV imaging, Scotland) at 30–35 d post-insemination. According to the results of the ultrasound examination, cows were grouped into two groups: non-pregnant (*n* = 194) and pregnant (*n* = 87). Then data for each cow from day of estrus till 7 days post estrus were acquired from the automated milking system concerning the following biomarkers: mP4e, MY, BHB, LDH, and BCS. The cows were being fed with total mixed ration (TMR) throughout the year at the same time, balanced according to their physiological needs. Cows were fed a TMR consisting of 50% grain concentrate mash, 25% corn silage, 15% grass silage, 5% grass hay, and 5% of mineral mixture. TMR was formulated accordingly to meet or exceed the requirements of a 550 kg Holstein cow producing 40 kg/d. Composition of ration: dry matter (DM) (%) 49,00; acid detergent fiber (% of DM) 20.00; neutral detergent fiber (% of DM) 28.00; non-fiber carbohydrates (% of DM) 39.00; crude protein (% of DM) 16.00; net energy for lactation (Mcal/kg). Feeding took place every day at 05:00 and 17:00. The cows were milked with DeLaval milking robot (DeLaval Inc., Tumba, Sweden). The average weight of the cows was 550 kg +/− 35 kg. Milk production during the year 2020 was 12,000 kg per cow and per year on average. 

### 2.2. Measurements

In order to gather data about mP4, MY, BHB, and LDH, the real-time analyzer Herd Navigator (Lattec I/S, Hillerød, Denmark) was used together with a DeLaval milking robot (DeLaval Inc., Tumba, Sweden). An inline sampler automatically took a representative sample of several milliliters of milk from each cow during the robot-milking process. The sample was then transferred into the Herd Navigator^TM^ for further analysis. BCS was measured with 3D BCS cameras (DeLaval body condition scoring BCS, DeLaval International AB, Tumba, Sweden).

#### 2.2.1. Measurement of mP4, MY, BHB and LDH

Novel real-time analyzer Herd Navigator™ (Lattec I/S, Hillerød, Denmark) was combined with DeLaval milking robot (DeLaval Inc., Tumba, Sweden) to detect milk BHB and LDH concentrations. During the milking process, a sample of several milliliters of milk was taken from each cow with the help of an inline sampler in order to determine the concentrations of before-mentioned parameters. The raw measurements were corrected according to company specified methods to take into account differences between sets of dry sticks and variations in surrounding humidity. Then, the most extreme outliers were removed from the calculations. Measurements over 200 µmol/min per liter were set to 200—a maximum value, and all negative values were taken out of the equation, because they did not meet with the normal range of measurements acquired by the Herd Navigator system. This is how data are normalized in the Herd Navigator system. Milk yield from each cow was measured using an optical milk meter. The LDH concentration (µmol/min) was calculated as the LDH activity divided by the milk yield from the latest milking activity.

#### 2.2.2. BCS Recording System

The technology behind body condition scoring is based on a 3D-camera which records certain parts of the animal: from above, the rear part of the back from the short ribs to the tail end. Every time a cow walks under the camera, the system identifies the specific movement and captures images of the cow; it then chooses the best image of the cow in the video-recording. The 3D-camera uses light coding technology, which projects a pattern of infrared ray dots on the back of the cow. Following that, the distances between these certain dots are measured; according to the manufacturer, a 3D image of the back is modeled, and an algorithm converts that image information into a body condition score. Since the cameras were placed above separation gates near the milking robot parlor, the cow’s measurements were taken every time they went to be milked. As a golden standard, the scale used to develop the algorithm was based on the visual scoring scale by Windman [15], a 1–5 point scale system. In this scale, the spinous to transverse processes are assessed and given a specific score where one corresponds to the lowest and five to the highest condition score. 

### 2.3. Data Analysis and Statistics

The cows (*n* = 281) had an average of 2.1 ± 0.1 lactations at 151.7 ±0.06 days postpartum. The average value of cow’s milk yield (MY) was 31 ± 0.02 kg/day, BHB—0.06 ± 0.001 mmol/L, LDH—27.3 ± 0.9 μmol/min, mP4—15.5 ± 0.7 ng/mL. The average BCS in cows, assessed on a five-point scale (from 1 to 5), was 3.2 ± 0.02 points.

Each variable from Herd Navigator^TM^ was grouped into two classes based on their arithmetic mean. MY values were divided into two classes: ≤ 31 kg/day (MY 0—52.3% cows) and > 31 kg/day (MY 1—47.7% cows), BHB values in milk—≤ 0.06 mmol/L (BHB 0—85.9% cows) and > 0.06 mmol/L (BHB 1—14.1% cows), milk LDH values—≤ 27 μmol/min (LDH 0—63.9% cows) and >27 μmol/min (LDH 1—36.1% cows), milk progesterone values—≤ 15.5 ng/mL (mP4 0—48.3% cows) and > 15.5 ng/mL (mP4 1—51.7% cows), BCS—≤ 3.2 (BCS 0—54.4% cows) and > 3.2 (BCS 1—45.6% cows). For a detailed assessment using Chi2 and binary logistic regression, the cows BCS data were grouped into five groups: (1) BCS > 2.5 (5.7% cows), (2) BCS = 2.5–3.0 (29.9% cows), (3) BCS = 3.0–3.5 (47.3% cows), (4) BCS = 3.5–4.0 (14.6% cows), and (5) BCS = 2.5–3.0 (2.5% cows). By lactation, the cows were divided into groups: lactation 1 and lactation ≥ 2 (L 1—58.0% cows). According to their pregnancy status, cows were divided into groups: non-pregnant (*n* = 194 or 69.0%) and pregnant (*n* = 87 or 31.0%) cows. The average DIM value in pregnant cows was 151.6 ± 0.11 days and in non-pregnant cows—151.7 ± 0.08 days.

The data from Herd Navigator^TM^ are presented as mean ± standard error (M ± SEM) and 95% confidence interval (CI). The normality of all variables was assessed by Shapiro–Wilk normality test. The *t*-test of independent samples was used to compare the difference in means between groups of pregnant and non-pregnant cows.

The chi-square (χ2) statistic was used to test the relationship between categorical variables—class of BCS and cow reproductive status, BCS and other indicators from Herd Navigator™.

To analyze factors contributing to the reproductive success of cows, multivariable logistic regression models were used, applying a backward stepwise logistic model to eliminate all non-significant explanatory variables. Categorical variables (lactation, BCS, MY, LDH, BHB, mP4) were continuously removed from the models according to the significance of the Wald criterion. 

Herd Navigator^TM^ data were analyzed using IBM SPSS software (version 26.0, IBM, Munich, Germany).

## 3. Results

### 3.1. Influence of Cows Pregnancy on Biomarkers

Of all the biomarkers, three differences between groups were significant. The BCS of the pregnant cows was higher (+0.49 score) and the MY was lower (−4.36 kg), and mP4 in pregnant cows was (+6.11 ng/mL) higher compared to the group of non-pregnant cows (*p* < 0.001). The data are presented in Table 1.

**Table 1:** Means and standard errors of the mean of biomarkers based on the pregnancy status of cows.

Biomarker	Pregnancy Status	M	SEM	95% CI	
				Lower Bound	Upper Bound
BCS, points	Non-pregnant	3.04	0.023	2.991	3.083
	Pregnant	3.53 ***	0.035	3.459	3.598
	Clinical thresholds [15]	1–5			
MY, kg/d	Non-pregnant	32.79	0.614	31.581	33.999
	Pregnant	28.43 ***	0.931	26.592	30.257
BHB, mmol/L	Non-pregnant	0.06	0.001	0.056	0.059
	Pregnant	0.06	0.001	0.055	0.059
	Clinical thresholds [5]	<0.080			
mP4, ng/mL	Non-pregnant	13.84	0.751	12.358	15.317
	Pregnant	19.95 ***	1.139	17.703	22.187
	Clinical thresholds [16]	7.6–20.4			
LDH, µmol/min	Non-pregnant	27.92	1.088	25.782	30.067
	Pregnant	25.62	1.649	22.372	28.866
	Clinical thresholds [6]	18.5–31.4			

*** *p* < 0.001. BCS—body condition score; MY—milk yield; BHB—milk β-hydroxybutyrate; mP4—milk progesterone; LDH—milk lactate dehydrogenase. M—mean; SE—standard error of the mean.

**Figure 1:**
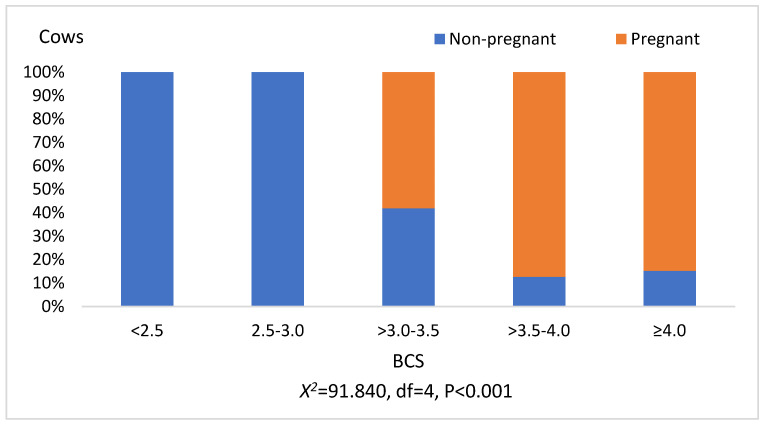
Relation of BCS score with pregnancy status of cows. BCS—body condition score.

As we can see from the first figure, the pregnancy status of the cows was associated with their BCS assessment (*p* < 0.001). BCS of pregnant cows was rated at three points (Figure 1). 

In the class of cows with BCS ≥ 3.0–3.5, 38.3% of cows were pregnant, whereas with BCS ≥ 3.5–4.0 75.6%, and with BCS > 4.0, 71.4% of cows were pregnant. 

### 3.2. Relationship of BCS with Other Indicators from Herd Navigator^TM^

**Figure 2:**
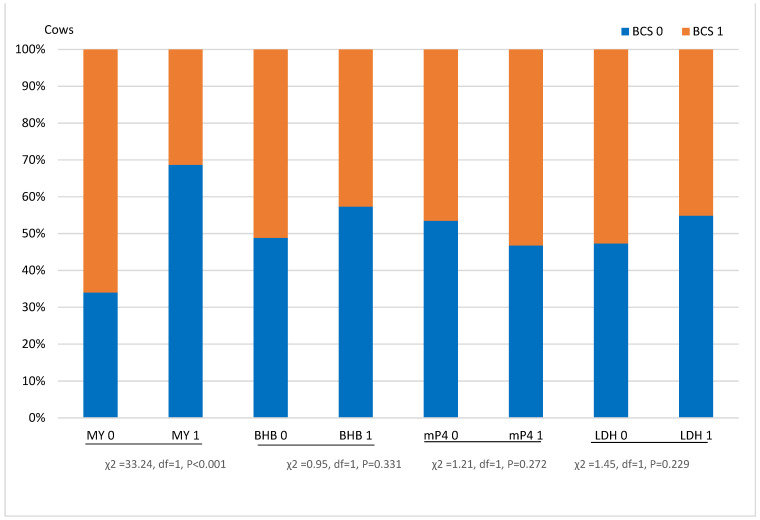
Relation of body condition score of cows with other biomarkers from the Herd Navigator^TM^.

The analysis showed that BCS was statistically significantly associated with other biomarkers (Figure 2).

MY 0—milk yield ≤ 31 kg/day; MY 1—milk yield > 31 kg/day; BHB 0—β-hydroxybutyrate in milk—≤ 0.06 mmol/L; BHB 1—β-hydroxybutyrate in milk > 0.06 mmol/L; LDH 0—milk lactate dehydrogenase ≤ 27 μmol/min; LDH 1—milk lactate dehydrogenase > 27 μmol/min; mP4 0—milk progesterone ≤ 15.5 ng/mL; mP4 1—milk progesterone > 15.5 ng/mL; BCS 0—body condition score ≤ 3.2 points; BCS 1 > 3.2 points. 

In the MY 0 class, cows with BCS > 3.2 accounted for 61.9%; in the MY 1 class—27.6% (*p* < 0.001). In the first BHB class, 8.3% fewer cows with BCS > 3.2 were determined, compared to class 0. The analysis showed that 6.7% more cows with BCS > 3.2 were found in the first mP4 class 1 than in class 0, and such cows in LDH class 1 were detected 7.4% less than in LDH class 0.

### 3.3. Relationship of BCS and Other Indicators from Herd Navigator^TM^ with Cows Pregnancy

The backward stepwise multivariate logistic regression showed that, of all tested categorical variables—lactation, BCS, MY, LDH, BHB, and mP4, only BCS had a significant effect on reproductive status of cows. We estimated that cows with BCS > 3.2 were 22 times more likely to have reproductive success than cows with BCS ≤ 3.2 (OR = 21.59, 95% CI = 10.381–44.903, *p* < 0.001).

Using the five BCS classes in the binary logistic regression analysis, we found that, with an increase in BCS of 0.5 points, the cow’s reproductive success increased eight-fold (OR = 7.952, 95% CI = 4.562–13.858, *p* < 0.001).

## 4. Discussion 

In this study, we acknowledged that mP4 in pregnant cows was higher compared to the group of non-pregnant cows. mP4 is considered a top indicator for the evaluation of reproduction status considering research purposes [16]. Consistently measuring progesterone concentrations also adds value to identifying pregnancy losses associated with luteal regression [17]. In addition to being more sensitive to metabolic changes (e.g., negative energy balance) in the early postpartum period than multiparous cows, primiparous cows are more likely to develop uterine diseases, which are known factors affecting resumption of postpartum cyclicity [18]. Data for mP4 have been widely used to characterize pregnancy status and ovarian activity and to evaluate associations between progesterone levels around the time of artificial insemination and pregnancy [16]. Eleven to fifteen days post insemination, a significant increase in progesterone concentrations can be registered, which indicates that the insemination has been a success [16]. Results of our study agree with Larson et al. [19], who noted that progesterone concentrations 5–10 days after insemination were higher in pregnant cattle. We found that in first seven days after insemination mP4 concentration was higher in pregnant cows, compared with non-pregnant.

Evaluation of BCS is a useful management tool to assess body fat stores of Holstein dairy cows [20]. In our study, we determined that cows from the pregnant group scored higher compared to cows in the non-pregnant group. Many studies have reported the relationship between BCS and reproduction in dairy cows. Cow health performance is strongly associated with BCS. For example, metabolic disorders, infertility, and lameness rates increase when cow condition deviates from the recommended BCS norms [21]. Changes in body condition score during peripartum have an effect on health and fertility of cows. Cows that lose more body mass are more prone to develop diseases during lactation. Higher BCS loss, therefore higher weight and fat adipose tissue loss, during the dry period increases BHB concentrations. Cows with increased BHB concentrations have lower pregnancy success at first artificial insemination compared to healthy cows. Greater number of inseminations per pregnancy, shorter activity at estrus, longer interval from calving to first observed estrus, and prolonged days open for cows with ketosis than are noticeable when compared to healthy cows [22]. In turn, higher circulating BHB concentration prolongs the period until first postpartum ovulation [23]. Monitoring of body condition score has received considerable attention as a tool to help with nutritional program managing in dairy herds [24]. Ketonemia, in turn, causes insulin resistance in dairy cows consistent with studies linking high BCS to reduced peripheral insulin sensitivity in the lipomobilization state [25]. BCS at calving is one of the most important factors linked to the timely resumption of fertile ovulation postpartum [26]. Changes in BCS in the last trimester of pregnancy are of lesser importance in determining re-conception postpartum in beef cows [26]. The results of our study concur with the findings of Graham, which state that cows with moderate to good BCS at calving have a higher re-conception rate than cows with poor BCS at calving [27]. In addition, the same author reports that cows with low BCS at calving have lower fertility [27]. This may be because cows that calved with a BCS of 3.0–3.5 had higher blood concentrations of insulin and glucose than cows with a BCS of 2.0–2.5 [15]. Other authors have reported that, in cows, BCS at parturition is the most important factor that determines the period to re-conception postpartum [28]. BCS and its changes seem to affect reproductive performance because they are indicators of the degree of negative energy balance [29]. Britt [30] suggested that negative energy balance (NEB) during folliculogenesis could impair follicular development and subsequent reproductive performance. According to the results of our study, we found that pregnant cows had 0.49 points higher body condition score than non-pregnant cows, and cows with BCS > 3.2 were 22 times more likely to have reproductive success than cows with BCS ≤ 3.2. Additionally, we found that with an increase in BCS of 0.5 points, the cow’s reproductive success increased eight-fold. 

We found that MY was lower in pregnant cows compared to non-pregnant. In literature, we found different results concerning the relationship of milk yield and reproductive performance of cows. According to some literature, there is no antagonism between daily milk yield and first postpartum insemination, and it does not affect the pregnancy rate from first insemination at the cow level; however this antagonism appeared at herd level, especially in herds where fertility was considered good (i.e., 50% herd probability of pregnancy at first AI). The magnitude of the relationship between milk yield and reproductive performance is small, and it depends on the level of herd production [31]. According to Buckley et al., [32] a high milk yield at first service was indicative of increased likelihood of being pregnant by 42 d of the breeding season. Authors Fulkerson [33] and Moate and Harris [34] show a positive relationship between milk production and reproduction (submission and conception rates). Other studies found no relationship between milk production and reproduction [35,36]. However, most of the studies have found an antagonistic relationship between milk production and several fertility traits [37,38,39]. These findings meet with the results of our study—pregnant cows had 4.36 kg/day lower milk yield than non-pregnant cows.

## 5. Conclusions

According to the aim of our current study, which was to determine the relation of biomarkers such as BCS, BHB, MY, LDH, and mP4 with pregnancy success in cows, we assessed that the group of pregnant cows had 0.49 points higher body condition score, 4.36 kg/day lower milk yield, and 6.11 ng/mL higher mP4 concentration than the group of non-pregnant cows. Pregnant cows had 0.49 points higher body condition score than non-pregnant cows, and cows with BCS > 3.2 were 22 times more likely to have reproductive success than cows with BCS ≤ 3.2.

## Data Availability

The data presented in this study are available within the article.
